# Chicago Biological Society

**Published:** 1882-03

**Authors:** 


### Society Reports.

### ARTICLE VIII.

CHICAGO BIOLOGICAL SOCIETY. Stated Meeting, February 1, 1882. Dr. C. Fenger, President, in the Chair.

The President presented a report of six cases of aneurisms, an abstract of which follows:

I.—The first was a case of traumatic and popliteal aneurism, in a man æt. thirty-five. On the Fourth of July he was shot in the left popliteal region. The wound was small, and caused but little hæmorrhage, but there was severe pain and difficulty in walking. On examination, the popliteal space was found swollen, there was the ordinary pulsation of aneurism, and a bruit was audible.

July 5 the patient was anæsthetized, an Esmarch's bandage 1olled up the thigh, and Dr. Fenger made an incision into the popliteal space, following a probe which had been introduced into the bullet wound as far as the anterior head of the gastrocnemius. The ball, caliber 32, was removed, and it was found that the popliteal artery had been perforated twice. This having been ligated, the wound was dressed antiseptically. The whole operation had been done according to Lister's method.

July 6, the temperature was 102.1, and moist gangrene set in. Dr. Fenger proposed amputation at middle of the thigh, but the patient refused, and gangrene extended upward.

July 8, amputation was made at the upper third of the thigh, but gangrene continued in the stump. Patient died at nine P. M. the 10th.

Professor Fenger said it was conservative surgery to apply a ligature as he had done in this case; that is, it agreed with the latest works on surgery, although immediate amputation was recommended by a few. Cases like the latter rarely proved successful. It has been advised to ligature the femoral artery, and the femoral vein also, in order to avoid gangrene. The doctor believed a simultaneous ligature of both vessels was advisable, but he could not explain why gangrene would be thus less likely to occur, although such was the case.

II.—The second case was also that of a man, and involved the left popliteal artery. March 17, 1880, the patient applied for treatment, the aneurism having increased for six weeks previous. Dr. Lee tied the femoral artery, and the patient did well, but on June 15 the aneurism returned. An elastic bandage was applied, but the tumor enlarged till July 20, when it was seven inches in length.

Dr. Fenger then operated antiseptically, and a dark fluid was removed from the sac of the aneurism, whose walls were those of the popliteal space. There was profuse hæmorrhage. He ligatured the popliteal artery, checking capillary hæmorrhage with a solution of chlorate of zinc, and introduced a drainage tube.

September 20, the aneurism had healed.

October 19, patient was discharged.

March 25, 1881, the extension of the limb was not perfect, on account of the large cicatrix, and there was some œdema about the foot; patient otherwise well.

III.—Aneurism of external iliac. Patient, a man, twenty-five years of age, had a neuralgia in his left foot for a year previous to April 20. On the latter day, while pulling on his boot, he felt some pain about Poupart's ligament, and soon a pulsating tumor made its appearance in the left groin. On examination, it was found to be three inches long and two inches wide, and was accompanied with the ordinary bruit. July 10, dry gangrene attacked some of the toes. Incision was made July 12, and the external iliac was ligatured. July 14, the gangrene had reached the malleoli. July 20, the wound in the groin had healed, but gangrene had advanced to the knee, and some hæmorrhage took place. July 26, hæmorrhage increasing, Dr. Fenger ligatured the common iliac. There followed much pain, delirium, and gangrene advanced above the knee. No line of demarkation had formed. Patient died the 29th.

*Autopsy.*—A clot had formed in the left common iliac; the catgut ligatures were absorbed, all but the knots, and arteritis deformans had set in in the arteries. Calcareous changes had taken place in the profunda femoris.

Complete recovery was not to be hoped for in cases like the above. Amputation had been justifiable after a line of demarkation had formed.

IV.—Aneurism of the head. Patient; male; æt. twenty-six. When a little boy, a small tumor formed at the anterior fontanelle. When fourteen years old, the tumor, then the size of a walnut, ruptured, and healed again, but the aneurism enlarged till he was twenty years old, when it was the size of the hand. It increased afterward, giving rise to little inconvenience. July 2, 1879, it ruptured, and a profuse hæmorrhage took place. August 2, the right common carotid was ligatured, with improvement in the aneurism. August 17, the left common carotid was also ligatured; patient stopped breathing, but afterward revived. Aphasia and difficulty in deglutition followed. August 20, extirpation of the aneurism. August 21, transfusion of blood into the patient. August 24, temperature was 104, and ulceration was taking place in the wound. August 25, patient died, with a temperature of 106. No autopsy. Septicæmia had caused death. This had been an unusually desperate case, especially on account of the spontaneous rupture of the aneurism.

V.—Traumatic aneurism of the carotid artery. The patient, a man, was shot in the cheek, near the margin of the orbit. On examination, Dr. Lee found the jaws closed, difficult deglutition, and swelling of the tonsils. Eight days afterward the patient was able to go out, but there was much pain in the right side of the neck; tonsillitis. A swelling appeared on the hard palate, which the doctor thought might be the ball. On opening into it, it proved an aneurism. Much hæmorrhage followed, and the patient died on the spot.

*Autopsy.*—Dr. Fenger, on opening the neck, found pus at the bifurcation of the carotids, some old and also some recent clots of blood, and an inch above, the artery had been perforated by the ball, caliber 32, which was found impacted in the sphenoid bone. This case was one of rare occurrence, and difficult in its diagnosis.

VI.—Aneurism of upper part of vertebral artery.

G. K., æt. nineteen, was shot in the back of the neck January 6, 1881, with a 32 caliber ball. There was swelling in the parotid region. The wound was two inches behind the mastoid process, aud severe pain was felt at the left angle of the lower jaw. January 11, the wound was dressed, and some pulsation was observed. January 15, a tumor made its appearance on the outside. Dr. Lee ligatured the left common carotid artery. Patient's temperature at one time reached 103, but improvement afterward followed.

February 1, patient appeared in court, had to talk a good deal, and returned to the hospital much worse. Pulsation returned behind the left mastoid process of temporal bone, and iodide of potassium, with ergot, were administered, but the aneurism increased. February 9, Dr. Fenger tried to ligature the arteries supplying the tumor, together with other tissues, but he had to make a free incision two and a half inches long, much hæmorrhage following. Splinters of bone were felt at the bottom of the wound. He removed the upper fourth of the sterno-cleido-mastoid, found that the aneurism was supplied by the vertebral artery, and ligatured the latter. Artificial respiration had to be resorted to, and eight ounces of defibrinated blood transfused into the patient. Pulse was 150 a minute. Patient rallied. February 17, the drainage tube was removed. Patient was well March 15, but a full motion of the head did not return until August.

In order to diagnosticate an aneurism of the vertebral artery, it was necessary to compress the latter over the carotid tubercle of the cervical vertebra. External compression of the artery, in one reported case, ended in a cure of the aneurism.

Prof. Fenger advised, as a conclusion of his paper, an early ligature of the arteries and removal of aneurisms. He did not believe in acapuncture or galvano-puncture, and thought that coagulation thus induced was not always desirable.

Dr. B. Bettman read an inaugural paper on "The Condition of the Eyes in two fatal cases of Pernicious Anæmia," an observation made by him in the Pathological Institute of Heidelberg.

I.—The first case was that of a female, æt. thirty-seven, whose blood, in June, 1880, contained 1,298,600 red corpuscles in each cubic millimeter. It contained also microcites and porculocites in large numbers. She died August 25. The appearance of the eyes was this: both papillæ were pale, the fundus was paler yet, and venous pulsation was observed on the papillæ. Small retinal hæmorrhages appeared in the right eye July 15. July 23, new hæmorrhages appeared, while the old ones had disappeared. They now made their appearance in the left eye, and could be referred to repeated vomiting. Under the microscope the stump of the optic nerve appeared normal, the retina was œdematous, and transudation had taken place in it. The axis fibers of the nerves were œdematous. The membrana limitans had been perforated at the seat of the hæmorrhages, and the rods and cones destroyed. Clots had formed between the retina and the choroid, probably at the time that vomiting had taken place.

II.—The second case, J. P., æt. forty-four, had been sick since the 14th of May. Spleen and liver enlarged. Painful spots were felt over the bones. Died June 25.

Examination of the eyes: Extravasation and hæmorrhages took place; white plaques appeared and disappeared; hydrops of the sheath of the left optic nerve was found, together with hæmorrhages, as in the preceding case. The right eye was similarly affected. There was serous transudation, especially in the neighborhood of the papilla. Varicose nerve fibers extended into the vitreous humor.

The preceding are but a few notes from the doctor's paper, which was a brilliant and masterly contribution to the disease under consideration. He was elected a member.

---

THE spray has been abandoned by many surgeons, and even Mr. Lister has spoken in qualified terms of its necessity; and had we to prophesy instead of to record accomplished facts we might venture to predict an early abandonment of this cumbrous addition to a surgeon's armamentarium.—*Med. Press and Circular.*

